# Action Generative Networks Planning for Deformable Object with Raw Observations

**DOI:** 10.3390/s21134552

**Published:** 2021-07-02

**Authors:** Ziqi Sheng, Kebing Jin, Zhihao Ma, Hankz-Hankui Zhuo

**Affiliations:** School of Computer Science and Engineering, Sun Yat-sen University, Guangzhou 510006, China; shengzq@mail2.sysu.edu.cn (Z.S.); jinkb@mail2.sysu.edu.cn (K.J.); mazhh7@mail2.sysu.edu.cn (Z.M.)

**Keywords:** AI planning, contrastive learning, action model

## Abstract

Synthesizing plans for a deformable object to transit from initial observations to goal observations, both of which are represented by high-dimensional data (namely “raw” data), is challenging due to the difficulty of learning abstract state representations of raw data and transition models of continuous states and continuous actions. Even though there have been some approaches making remarkable progress regarding the planning problem, they often neglect actions between observations and are unable to generate action sequences from initial observations to goal observations. In this paper, we propose a novel algorithm framework, namely AGN. We first learn a *state-abstractor model* to abstract states from raw observations, a *state-generator model* to generate raw observations from states, a *heuristic model* to predict actions to be executed in current states, and a *transition model* to transform current states to next states after executing specific actions. After that, we directly generate plans for a deformable object by performing the four models. We evaluate our approach in continuous domains and show that our approach is effective with comparison to state-of-the-art algorithms.

## 1. Introduction

For future robots to perform general tasks in unstructured environments such as homes or hospitals, they must be able to reason about their domains and plan their actions accordingly. In AI literature, this general problem has been investigated under two main paradigms—automated planning and scheduling [1] (AI planning) and reinforcement learning [2]. At the same time, many objects in human daily life are deformable or nonrigid, such as clothes and ropes. Hence, dealing with deformable objects planning is a significant issue. In this issue, there have been many studies that seek to handle deformable object planning problems [3,4,5].

Researchers face two main challenges when handling deformable object planning tasks. On the one hand, unlike strict objects planning tasks, it is often difficult to specify logical representation of a state correctly in deformable object related domains. For example, considering designing a logical representation of the state of a deformable object such as a cloth, it is difficult to “logically” specify features of the deformable objects, e.g., bending angles, relative positions of different parts of deformable objects, etc. On the other hand, the action models of deformable things are sophisticated and nonlinear [6], which makes modeling and completing planning task in such deformable object domains challenging.

One category of studies managing the challenges in continuous states and actions domains is model-free learning [7,8]. They either relied on domains whose rewards are instrumented [9,10,11], or required high-quality demonstrations to guide the learning process [12]. Without high-quality demonstrations, however, model-free learning is notoriously weak, and often needs huge numbers of instances to learn from.

Another category of studies, i.e., model-based learning, has also shown promising in sample-efficient learning [13,14]. Using such model-based learning studies for deformable objects, however, researchers should consider how to represent state and learn action models appropriately. Some approaches take a direct approach to learning complex action models through raw space [4,15]. However, compared with latent space, raw space has too much redundant information, which is not conductive to model learning. The other approaches, such as Agrawal et al. [16] and Nair et al. [17], aim to learn forward dynamic models for manipulating deformable objects. Other model-based studies such as thanard et al. [18] train Causal InfoGANS [19] to both obtain visual representations and action models for planning. However, those techniques are weak due to training instabilities concerned with GANS [20] and cannot generate actions to guide robot to perform tasks.

In this paper, we propose a novel model-based algorithm framework, called AGN, which stands for action generative network, to compute action sequences for guiding an agent to perform a task from initial observations to target observations, and predict updating observations after executing actions. AGN uses contrastive technology to learn both the underlying heuristic models and transition models for deformable objects at the same time. We assume that using contrastive technology for model-based learning obtains better generalization and latent space structure with its inherent information maximization loss function. We modified the loss function posed in contrastive predictive coding [21] to learning effective transition model and heuristic model jointly. When the latent models for representations, the transition model, and the heuristic model are learned offline, we can use these models to manipulate deformable objects from a certain initial observation to the desired goal observation.

## 2. Related Work

### 2.1. Deformable Object Planning

There has been a lot of work in the area of robotic manipulation of deformable objects [22]. The deformable object handling problem has been studied via classical methods such as motion planning and manipulation [23]. There has been recent interest in combining deep generative models with structured dynamical systems in the context of variational autoencoders, where the latent space is continuous [24]. Watter et al. [25] used such models to perform the planning via learning latent linear dynamics and exploiting a linear quadratic Gaussian control algorithm. Causal InfoGAN [18] used Gumbel-Softmax to backprop through transitions of discrete binary states, and leveraged the structure of the binary states for planning. Ha et al. [26] presented a representation learning algorithm that learned a low-dimensional latent dynamical system from high-dimensional sequential raw data, e.g., videos.

In the planning literature, most studies relied on manually designed state representations. In a recent work, Konidaris et al. [27] automatically abstracted state representations from raw observations, but relied on a prespecified set of skills for the task. Sriniva et al. [28] introduced universal planning networks that embedded differentiated planning within a goal-directed policy. This planning computation unrolls a forward model in a latent space and infers an optimal action plan through gradient descent trajectory optimization. The plan-by-gradient-descent process and its underlying representations are learned end-to-end to directly optimize a supervised imitation learning objective. Our approach performs a goal-directed deformable planning by using the linear interpolation method and can achieve convergence quicker than other methods.

### 2.2. Contrastive Prediction

It remains a challenge to learn a valid representation in the deformable object domain. Many researchers seek to use contrastive learning methods to handle this problem. For example, Word2Vec [29] optimizes a contrastive loss to demonstrate semantic and syntactic structure in the learned latent space for words. Oord et al. [21] introduce negative examples to learn abstract representations of high-dimensional data, for example, pictures. Tian et al. [30] learn abstract representations by letting different views of images be embedded closely to another, and further from the others through a contrastive loss. Lately, SimCLR [31] achieves good results in self-supervised learning representations, by introducing a nonlinear transformation between the representation and the contrastive loss.

Different from the above-mentioned work, we aim to consider generating action sequences by introducing action transition relations in AGN. Instead of directly planning on the high dimension observation, we choose to plan in low latent space. AGN perform a goal-directed planning process by using the heuristic model and transition model iteratively, which is useful and can converge quicker than other methods.

## 3. Our AGN Approach

In this section, we introduce our framework, AGN, which stands for action generative network for deformable object planning (AGN). We begin with presenting the problem formulation. After that, we address our AGN algorithm framework in detail. Finally, we describe the procedure of solving a goal-directed planning problem with AGN.

### 3.1. Problem Definition

**Figure 1:**
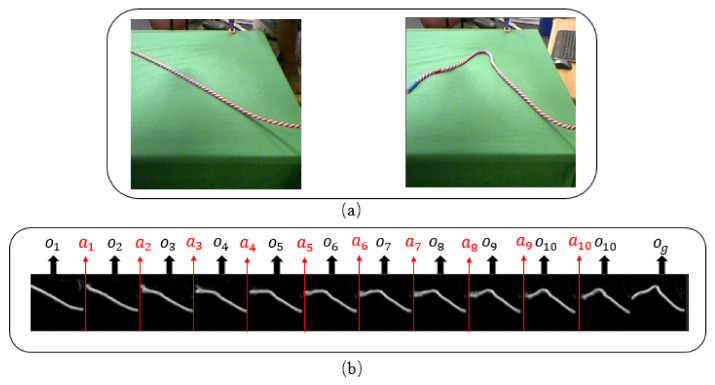
(**a**)Examples of initial observations and goal observations in rope domain. (**b**) Examples of 10 step trajectory, given the initial and goal observations in (**a**).

Our training data are a set of trajectories $T=\langle l_{1},l_{2},\cdots l_{n}\rangle$, each trajectory $l_{i}\in T$, is defined by $l_{i}=\langle o_{0},a_{1},o_{1},a_{2},\ldots ,a_{N-1},o_{N}\rangle$, where $o_{i}$ is a raw observation, e.g., image, and $a_{i}$ is an action denoted by a *N*-dimension tensor. Note that each dimension of an action has specific meaning. A raw observation $o_{i}$ is changed into a new raw observation $o_{i+1}$ after executing action $a_{i}$. For example, in the rope domain shown in Figure 1, an action is represented by a vector of five elements: $\left(p_{x},p_{y},\phi ,c,g\right)$, where $p_{x},p_{y}$ are x-coordinate and y-coordinate of the point of the rope where the action is executed. ϕ is the angle of the rope being moved by the action. *c* is the length of the rope moved by the action, *g* is a boolean value indicating whether the action should be used for training. As an example, action “(2.0,3.0,π,0.05,1)” indicates the point (2.0,3.0) of the rope is moved with π degrees and 0.05 meters length and the action will be used for training due to g=1.

We define our learning problem as: given a set of training data T, we aim to learn a planning model M, i.e., action generative networks, using T. With the learned M, we formulate our planning problem as a tuple $P=\langle M,o_{0},o_{g},A\rangle$, where $o_{0}$ is an initial observation, $o_{g}$ is a goal observation, A is a set of actions. We aim to solve the planning problem P by generating a trajectory (i.e., a plan) $\sigma =\langle o_{0},a_{0},\ldots ,a_{n-1}.o_{g}\rangle$ that transforms the initial observation $o_{0}$ to goal observation $o_{g}$.

An example of initial observation and goal observation is shown in Figure 1a, where the figure on the left side shows an initial observation and a goal observation is shown in the figure on the right side. Figure 1b is a plan of 10 actions transforming $o_{1}$ to $o_{g}$. Each action in the plan updates the positions of different points of the rope in different directions until reaching the goal.

### 3.2. Algorithm Framework

In this section, we introduce our proposed framework for learning deformable object manipulation from fully observable raw observations: action generative network (AGN). We begin with the details of our approach. After that, we discuss our planning process with AGN in the next section.

**Figure 2:**
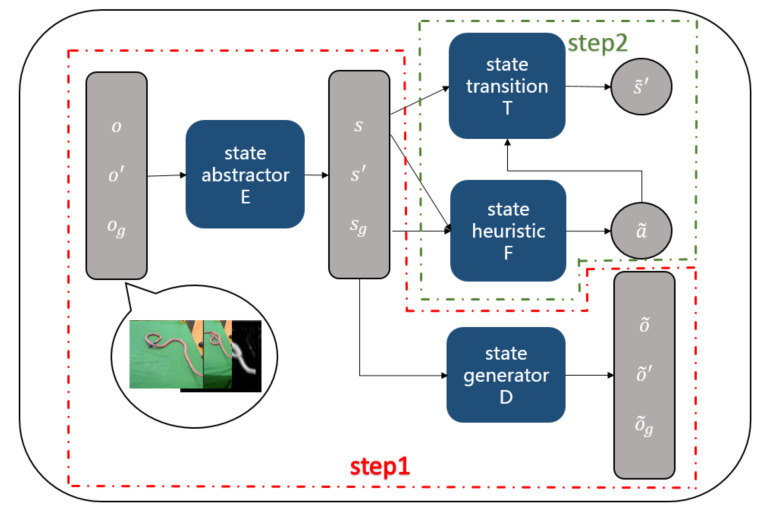
The framework of our action generative network (AGN).

An overview of our AGN approach is shown in Figure 2. The training process of our algorithm contains two steps: we first jointly train an auto-encoder, including a state-abstractor model $E(o;\theta_{1})=s$, which extracts the low-dimensional latent abstract state *s* given a raw observation *o*, and a state-generator model $D(s,z;\theta_{2})=o$, which generates a raw observation given a low-dimensional latent abstract state *s* and noise *z*. After that, we train a heuristic model $F\left(s_{t},s_{g};\theta_{3}\right)=a_{t}$, which generates action $a_{t}$ to be executed on state $s_{t}$ given current state $s_{t}$ and goal state $s_{g}$, and a transition model $T\left(s_{t},a_{t};\theta_{4}\right)=s_{t+1}$, which generates a new state $s_{t+1}$ given a current state $s_{t}$ and an action $a_{t}$. $\theta_{1}$, $\theta_{2}$, $\theta_{3}$ and $\theta_{4}$ are parameters of the four models, respectively, which are to be learned with the training data.

Planning in high-dimensional continuous domains is hard in general. Therefore, we consider planning based on low-dimension latent space. In order to learn the conversion between the high-dimension raw observation and low-dimension latent state, we first learn an auto-encoder, which contains a state-abstractor model $E(o;\theta_{1})=s$ and a state-generator model $D\left(s,z;\theta_{2}\right)=\tilde{o}$. The reason of adding noise into state-generator model is to improve the robustness. We jointly learn a state-abstractor model $E(o;\theta_{1})=s$ and a state-generator model $D\left(s,z;\theta_{2}\right)=\tilde{o}$ by minimizing the MSE loss comparing the real raw observation *o* with the reconstructed raw observation $\tilde{o}$, which is defined by Equation (1).

(1) $$\begin{array}{c} L={∥o-D(E\left(o;\theta_{1}\right))∥}_{2} \end{array}$$

**Figure 3:**
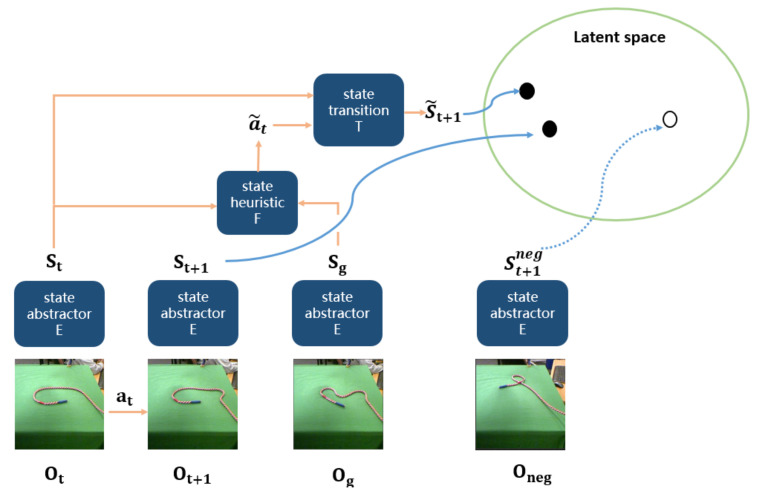
The framework of the joint-training heuristic model and transition model.

After learning the projection between high-dimensional raw observations and low-dimensional latent space, then we jointly learn the heuristic model *F* and the transition model *T*. The whole process of jointly training heuristic model *F* and transition model *T* is shown in Figure 3. Heuristic model *F* predicts an action ${\tilde{a}}_{t}$ given a current state $s_{t}$ and a goal state $s_{g}$ by Equation (2). Then transition model *T* updates current state $s_{t}$ to a next state $s_{t+1}$ after executing ${\tilde{a}}_{t}$ by Equation (3).

(2) $$\begin{array}{cc} {\tilde{a}}_{t} & =F(s_{t},s_{g};\theta_{3}) \end{array}$$

(3) $$\begin{array}{cc} {\tilde{s}}_{t+1} & =T(s_{t},{\tilde{a}}_{t};\theta_{4}) \end{array}$$

where ${\tilde{a}}_{t}$ is the predicted action, $a_{t}$ is the real action. $s_{t}$ is the current state, ${\tilde{s}}_{t+1}$ is a predicted next state, $s_{t+1}$ is the real next state, $s_{g}$ is a goal state. $s_{t}$, $s_{t+1}$, $s_{g}$ is computed by state-abstractor model given a current observation $o_{t}$, the next observation $o_{t+1}$, a goal observation $o_{g}$. Then we train the heuristic model with a loss function defined by Equation (4).

(4) $$L_{F}={∥a_{t}-{\tilde{a}}_{t}∥}_{2}$$

Next we define an InfoNCE contrastive loss described by Oord et al. [21], which is defined by Equation (6), where ${\dot{s}}_{t+1}=\langle {\dot{s}}_{t+1}^{0},\cdots ,{\dot{s}}_{t+1}^{k-1}\rangle$ is a set of incorrect latent states different from the real next state $s_{t+1}$. An incorrect latent state ${\dot{s}}_{t+1}^{i}$ is generated by a sample in a set of negative samples ${\dot{o}}_{t+1}=\langle {\dot{o}}_{t+1}^{0},\cdots ,{\dot{o}}_{t+1}^{k-1}\rangle$. We construct negative samples ${\dot{o}}_{t+1}$ by random selecting *k* samples, the latent state of each sample is different from the real next state $s_{t+1}$. The *h* function shown in Equation (6) is some similarity function between the computed latent states, which is computed by Equation (5). The motivation behind this objective function is to let the predicted states and their corresponding positive samples be close in latent space.

(5) $$\begin{array}{cc} h(z_{1},z_{2}) & =exp(-{∥z_{1}-z_{2}∥}^{2}) \end{array}$$

(6) $$\begin{array}{cc} L_{c} & =-E[log\frac{h({\tilde{s}}_{t+1},s_{t+1})}{\sum_{i=0}^{k-1}h\left({\tilde{s}}_{t+1},{\dot{s}}_{t+1}^{i}\right)}] \end{array}$$

Then we define an L2 norm of convariance matrix to full the loss L by Equation (7) following tharand et al. [18], aiming at learning a latent planning system such that linear interpolation between states makes for feasible plans. To bring about such latent space, we consider transition probabilities $T_{M}\left(s_{t+1}|s,a;\theta_{4}\right)$ given as Gaussian perturbations of the state: $s_{t+1}=s+\delta$, where $\delta ∼N(0,\Sigma_{\theta_{4}}\left(s\right))$, and $\Sigma_{\theta_{4}}\left(s\right)$ is a diagonal convariance matrix. The key idea here is that, if only small local transitions are possible in the system, then a linear interpolation between two states $s_{0}$ and $s_{g}$ has a high probability, and it represents that a feasible trajectory exists in the observation space.

(7) $$L_{n}=E_{s∼P_{M}}\left|\right|\Sigma_{\theta_{4}}\left(s\right){\left|\right|}_{2}$$

where the prior probability $P_{M}$ for each element of *s* is uniform in [−1,1].

Therefore, the loss function of transition model can be defined by Equation (8). Finally, we jointly learn the heuristic model *F* and transition model *T* by minimizing the loss function defined by Equation (9), where λ is a hyper-parameter.

(8) $$\begin{array}{cc} L_{T} & =L_{c}+L_{n} \end{array}$$

(9) $$\begin{array}{cc} L & =\lambda L_{F}+\left(1-\lambda \right)L_{T} \end{array}$$

### 3.3. Planning with *AGN*

After training the state-abstractor model *E*, state-generator model *D*, heuristic model *F*, and transition model *T*, naturally, we use them for planning to solve deformable object planning problems, aiming at computing an action observation trajectory to reach $o_{g}$ from $o_{0}$. The overall planning process can be divided into three steps.

- Firstly, state-abstractor model *E* outputs abstract state $s_{0}$ and $s_{g}$ with $o_{0}$ and $o_{g}$, respectively.
- Secondly, we compute an action sequence reaching $s_{g}$ from $s_{0}$ and derive an action state trajectory $\gamma =\langle s_{0},a_{0},s_{1},a_{1},\ldots ,a_{N-1},s_{N}\rangle$ by Algorithm 1. We first perform linear interpolation between $s_{0}$ and $s_{g}$, and attain an initial sequence η = $\left[s_{0},s_{1},\cdots ,s_{n},s_{g}\right]$. As for each pair of $s_{i}$ and $s_{i+1}$, we compute an action $a_{i}$ by the heuristic model. If $s_{i}$ can reach $s_{i+1}$ after executing action $a_{i}$, we add state $s_{i}$ and action $a_{i}$ into θ. Otherwise, we interpolate a latent state $s_{mid}$ into η between $s_{i}$ and $s_{i+1}$. We repeat the above procedures until each pair of states in η can be transformed by an action computed by the heuristic model. Finally, we attain an action state trajectory $\gamma =\langle s_{0},a_{0},s_{1},a_{1},\ldots ,a_{N-1},s_{N}\rangle$.

**Algorithm 1** planning algorithm.**input:**$s_{0}$, $s_{g}$, *F*, *T*.
**output:**
$\gamma =\langle s_{0},a_{0},s_{1},a_{1},\ldots ,a_{N-1},s_{N}\rangle$

1:do linear interpolation between $s_{0}$ and $s_{g}$, get η = $\left[s_{0},s_{1},\cdots ,s_{n},s_{g}\right]$2:i=0, γ=[]3:**while** i < n **do**4: $a_{i}$ = F(η[i],η[i+1]), ${\tilde{s}}_{i+1}$ = $T(\eta \left[i\right],a_{i})$5: **if** $\left|\right|{\tilde{s}}_{i+1}-\eta \left[i+1\right]{\left|\right|}_{2}<1e-3\;$ **then**6:  γ = [γ|γ[i+1]], $\gamma =[\theta |a_{i}]$7:  
i+=18: **else**9:  
$s_{mid}=\left(\eta \left[i\right]+\eta \left[i+1\right]\right)/2$10:  
$\eta =\eta \left[0:i+1\right]+\left[s_{mid}\right]+\eta \left[i+1:\right]$11:  
n+=112: **end if**13:
**end while**

- Finally, we compute an action observation trajectory $\sigma =\langle o_{0},a_{0},o_{1},a_{1},\ldots ,$$a_{N-1},o_{N}\rangle$. We first sample *k* different Gaussian noises randomly. Then we can obtain *k* different action observation trajectories given an action state trajectory θ and a noise by state-generator model *D*. At last, we select an optimal action observation trajectory $\sigma =\langle o_{0},a_{0},o_{1},a_{1},\ldots ,a_{N-1},o_{N}\rangle$ among the k trajectories.

Since the states and the actions for deformable object are in continuous space, the optimality and determinism of the solutions can hardly be discussed in this paper. In summary, given an initial observation and a goal observation, we can finally obtain a feasible trajectory that is valid and clear compared to other state-of-the-art methods.

## 4. Experiments

In our experiments, we aimed to (1) visualize the abstract states and planning in AGN; (2) show that AGN can produce realistic visual plans in a complex dynamical system; (3) show that AGN significantly outperforms baseline methods.

We began our investigation with a set of experiments in the rope domain, specifically designed to demonstrate the benefits of AGN, where we also compared AGN with other methods. We later present experiments on a real dataset of robotic cloth manipulation and verified the influence of two important hyper-parameters. Since both cloth and rope datasets are collected in the real physical environment, the final plan we learned is definitely fitted to a real setting.

### 4.1. Baselines

In order to evaluate AGN, we compared our approach with state-of-the-art algorithms. The first one is the visual forward model [32]; we achieve it by realizing training and planning process purely in pixel space. Secondly, we jointly learn a forward and inverse model following Lee et al. [16]. Finally, we compared AGN to causal InfoGAN [18], synthesizing plans to transit from initial observations to goal observations based on the InfoGAN [18] framework.

In consideration of the failure of the visual forward model and the causal InfoGAN to generate action sequences, we have trained an inverse model on the dataset, given a current observation $o_{t}$ and a next observation $o_{t+1}$, the action between $o,o_{t+1}$ can be generated.

### 4.2. Evaluation Criterion

We evaluate our approach based on three aspects:

- Trajectory confidence, to evaluate whether an observation transition is feasible or not.
- Trajectory distance, to evaluate the Euclidean distance between the current observation and the next observation after the current action is performed.
- Final-to-goal distance, to evaluate the Euclidean distance between the final observation and goal observation.

In order to quantitatively analyze the action trajectories we generated, we take the pretraining model proposed by Therand et al. [18], which is called Judge, to evaluate whether an observation transition is feasible or not. Trajectory confidence value is in [0, 1], a higher score represents a higher confidence coefficient. Given an output trajectory $\sigma =\langle o_{0},a_{0},o_{1},a_{1},\ldots ,a_{N-1},o_{N}\rangle$, we can compute the trajectory confidence used by the Judge in Equation (10).

(10) $$\begin{array}{c} tc=\frac{1}{N}\sum_{i=1}^{N-1}Judge\left(o_{i},o_{i+1}\right) \end{array}$$

where tc is trajectory confidence, *N* is the length of trajectory.

Moreover, we train a path distance function EVAL to evaluate the Euclidean distance between the current observation and the next observation after the current action is performed. Trajectory distance is computed by Equation (11).

(11) $$\begin{array}{c} td=\frac{1}{N}\sum_{i=1}^{N-1}EVAL\left(o_{i},a_{i},o_{i+1}\right) \end{array}$$

where td is trajectory distance and the less td is, the better a trace is, *N* is the length of trajectory.

We also compare the final-to-goal distance. Final-to-goal distance is the Euclidean distance between the final observation and goal observation, indicating that the smaller the final-to-goal distance is, the better action trajectory is.

Then we introduce the training process of the Judge model and EVAL model in detail:

1. The Judge model takes a pair of observations $\left(o_{t},o_{t+1}\right)$ as input and outputs a binary result of whether the observation is feasible or not. The training dataset consists of positive observation pairs, which are 1 timestep apart, and negative pairs that are randomly sampled from different rope manipulation trajectories. To avoid the background of rope influencing the training of Judge, we preprocess the rope data using the background subtraction pipeline mentioned above.
  To validate the accuracy of the Judge model, we evaluate it with observation traces to observe the binary outputs. Given an *m*-length observation trace, Judge takes the first observation and an observation, which is *n* steps apart, where *n* is from 1 to m−1. The binary output decreases from 1 to 0 smoothly with *n* increasing, indicating that the Judge model has the ability to recognize a feasible observation pair. We test Judge with 100 traces out of the testing dataset for AGN and the accuracy is 98%.
2. The EVAL model takes a pair of observations $\left(o_{t},{\hat{o}}_{t+1}\right)$, an action $a_{t}$, and an observation $o_{t+1}$ as inputs, where ${\hat{o}}_{t+1}$ is a predicting next observation and $o_{i+1}$ is a real next observation, they are updated from a current observation $o_{t}$ after executing action $a_{t}$. The EVAL model outputs a distance between $o_{t+1}$ and ${\hat{o}}_{t+1}$. The training dataset consists of positive next observations, we trained the EVAL model by letting the predict next observation ${\hat{o}}_{t+1}$ be close to the real next observation $o_{t+1}$. On a held-out test set, the distance between the predict next observation and real next observation converges to 0.

Note that the Judge and EVAL models are trained independent of AGN. Thus, trajectory confidence and trajectory distance are both impartial metrics.

### 4.3. Rope Manipulation

The rope dataset [17] contains sets of sequential pictures and corresponding actions, collected by a robot operating a rope in a self-supervised manner. The sample size used in the training process is 100,000. Each initial picture is 64×64×3 RGB. In order to remove interference factors, we converted the images to grayscale images, and used a model *BRM* proposed by Therand et al. [18] to remove the background, aiming at focusing on the object itself, which can avoid the algorithm overfitting to the background.

Regarding the definition of states, we follow the configuration of continuous abstract states specified in [18]. In this section, we intend to verify the effectiveness of the algorithm to handle deformable objects with continuous actions and continuous states.

**Figure 4:**
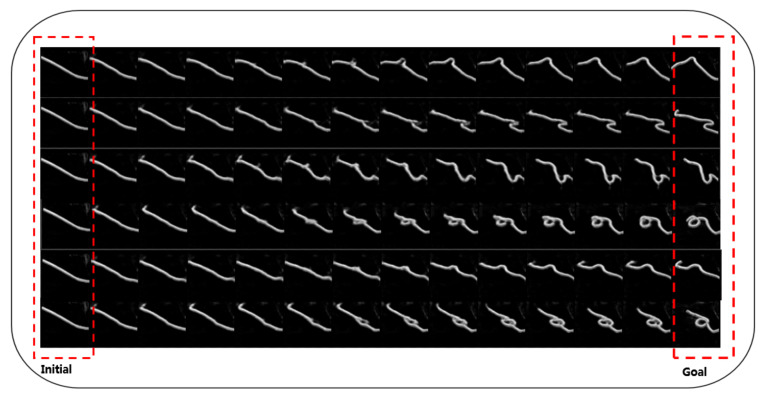
Result for rope manipulation data. The plot shows six planning instances, from left (initial observation) to the right (goal observation).

Table 1 shows the results of AGN and baselines in the rope domain. We trained on 800 pairs of test samples to obtain this average results. As shown in Table 1, our algorithm framework outperforms the other baseline in all kinds of evaluation methods, which verifies the reliability of our method. In term of trajectory confidence, it means that we can generate paths that are much more confident and much smoother than other algorithms. As for the trajectory distance, AGN is significantly lower than the others. Because the visual forward method and causal InfoGAN neglect actions, they cannot reason about the transition and updating between observations after executing actions. As for final-to-goal distance, AGN can generate action trajectories that are closer to the goal observation more effectively, which indicates that AGN outperforms the other three algorithms in goal-arrived tasks. Visual forward and joint dynamics are poor at long distance planning; therefore they are often unable to reach the goal. Figure 4 shows six examples generated by AGN and each row is a trajectory between initial raw observations and goal raw observations. As shown in Figure 4, given different pairs of initial and goal observations, AGN is able to generate a well-shifted and clear observation path.

### 4.4. Cloth Manipulation

**Figure 5:**
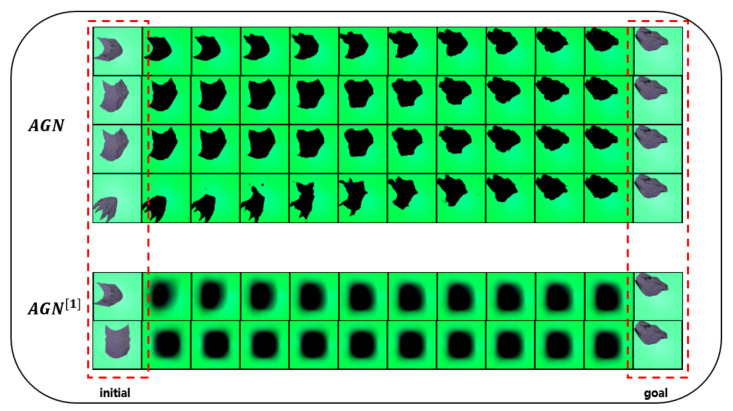
The first four rows are the origin algorithm **AGN**; the last two rows are $AGN^{\left[1\right]}$ training in raw observation space. Given initial observation and goal observation, **AGN** can attain valid trajectories.

In this section, we present the results of our experiments in the cloth domain to verify the effectiveness of our algorithm framework. The sample size used in the training process is 400k. Because training on the cloth domain is more difficult than training on the rope domain, we used a larger sample size. As shown in Figure 5, given a pair of an initial observation and a goal observation, we can finally obtain a valid trajectory. Since actions are abstract tensors, they do not have graphical representations. Then we compare origin AGN with AGN training in raw observation space. The last two rows of Figure 5 show that training AGN in raw observation space cannot learn correct action models, leading to bad trajectories.

**Figure 6:**
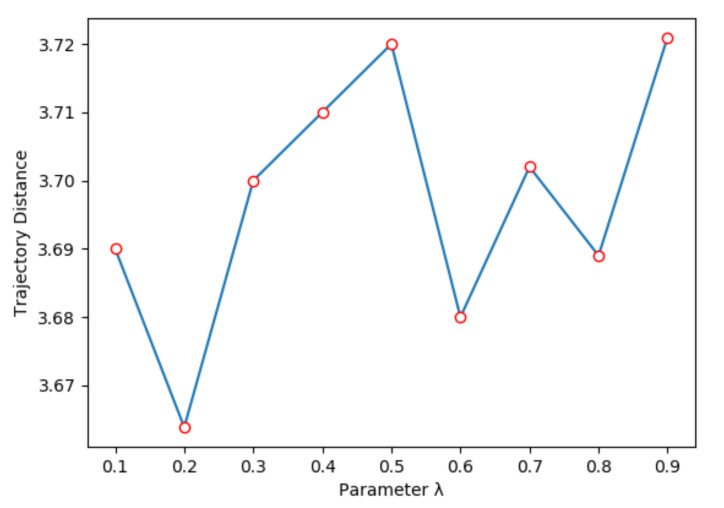
Trajectory distance with different parameter λ.

**Figure 7:**
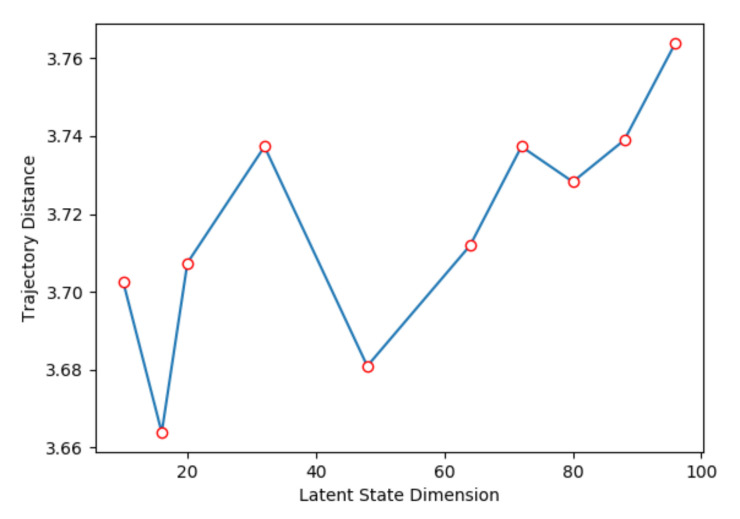
Trajectory distance with different latent state dimension.

We jointly trained the heuristic model and transition model; the ratio between heuristic model loss and transition model loss is λ, which is a hyper-parameter, shown in Equation (9). Figure 6 shows the relation between λ and trajectory distance. When λ=0.2, the trajectory distance is the smallest, because the heuristic model will inevitably have gradient flow when training transition model. Further, we also compare the performance with different latent state dimension. As shown in Figure 7, where we set λ=0.2, when the latent state dimension is 16, we can obtain the smallest trajectory distance. After that, the trajectory distance slowly grows as the latent state dimension increases, because it is hard to express all of the information in an observation with a low dimensional state vector; further, it becomes more difficult to train a neural network with more weights when the dimension size increases.

## 5. Conclusions

In this paper we propose a novel planning model learning framework, AGN, by considering actions between observations. Based on AGN, we learn four models, i.e., the state-abstractor model, state-generator, heuristic model, and transition model, and solve new planning problems with the learned models. Our experimental results show that our AGN approach is effective in comparison to baselines. In the future, we would like to extend our work to complex domains and consider objects in our framework that can better leverage the benefit of both deep learning and classical AI planning. It is also interesting to investigate the possibility of applying our AGN approach to learning action models [33,34,35,36] and recognizing plans [37,38,39] in the planning community.

## Figures and Tables

**Table 1:** Evaluation of planning result in rope domain.

	Trajectory Confidence	Trajectory Distance	Final–to–Goal Distance
visualforward	0.719	9.7189	5.5484
jointdynamics	0.567	10.680	5.046
ausalinfoGAN	0.884	9.0219	2.29
**AGN**	**0.935**	**1.432**	**2.126**

## Data Availability

Not applicable.
